# Middle school students’ psychological health on suicide ideation: based on latent profile analysis

**DOI:** 10.3389/fpubh.2024.1390682

**Published:** 2024-05-31

**Authors:** Meiting Wei, Jiang Liu

**Affiliations:** Faculty of Education, Yunnan Normal University, Kunming, China

**Keywords:** psychological health, suicide ideation, middle school students, section differences, latent profile analysis

## Abstract

**Introduction:**

This study identifies potential categories of mental health for adolescents in different school years and further analyzes the relationship between these categories and suicidal ideation.

**Methods:**

A total of 1944 middle school students completed SCL-90 and Self-rating Idea of Suicide Scale on November 29, 2022, selecting via a whole-group sampling method. Latent profile analysis was used to analyze the psychological health subtypes of students from a middle school in Southwest China. The R3step method and the DU3step method were conducted to analyze the predictive role of demographic variables and the effects of different profiles on suicidal ideation.

**Results:**

Different potential categories of psychological health were observed among middle school students. Junior middle school students can be classified into three types: Psychological Health Type (62.3%), Low-risk Type (27.1%) and High-risk Type (10.7%). Senior middle school students can be classified into four types: Psychological Health Type (43.3%), Low-risk Type (33.9%), Medium -risk Type (16.8%), and High-risk Type (6.0%). Gender and subjective family atmosphere are predictors of psychological health, and they also influence the population distribution of psychological health patterns in different sections of middle school students. Girls and students with poor subjective family atmosphere are more prone to experiencing psychological problems. There were significant differences in suicidal ideation among different potential categories of psychological health of different sections middle school students (*χ*^2^ = 1178.71, 1174.85, *p*<0.001). Among senior high school students classified as High-risk Type, they exhibited the highest score for suicidal ideation.

**Conclusion:**

There is obvious group heterogeneity in psychological health of different sections middle school students. Older students are more likely to have suicidal thoughts.

## 1 Introduction

Before 2020, over 700,000 people worldwide took their own lives annually, making suicide the fourth leading cause of death among individuals aged 15–29 (1), attracting worldwide attention. The spread of the COVID-19 pandemic has further exacerbated the global public health issue of suicide (2–4), with increased rates of suicidal ideation (10.81%), suicide attempts (4.68%), and self-harm behaviors (9.63%) reported in most countries and regions (4). Additionally, there has been a more significant deterioration in adolescent mental health and a greater increase in suicide rates (3, 5–7). Despite a significant reduction in the severity of the pandemic, concerns linger as the impact of COVID-19 on mental health may persist for a prolonged period (8). In recent years, while the suicide mortality rate in China has steadily declined, there has been a disproportionately sharp rise among adolescents (9, 10). Hence, the issue of adolescent suicide warrants continued emphasis and attention. Adolescent suicide generally involves two stages: the first stage involves the emergence and development of suicidal ideation, and the second stage is the process of transitioning from suicidal ideation to suicidal behavior (11). Suicidal ideation stands as one of the most critical risk factors for future suicide attempts (12), where suicidal ideation and attempts during adolescence can predict suicidal behavior in adulthood (13). A meta-analysis finding by Yu et al. revealed that suicidal ideation was notably prominent among junior and senior high school students in China from 2010 to 2020, surpassing that among college students, with higher detection rates observed among senior high school students compared to junior high school students (14). Therefore, strengthening attention toward suicidal ideation among middle school students and implementing early prevention, timely screening, and prompt intervention measures can help alleviate the formation of suicidal ideation and prevent its progression to suicidal behavior. In summary, investigating the influencing factors and mechanisms related to suicidal ideation among high school students not only provides empirical evidence for the prevention and intervention of adolescent suicide but also holds practical significance for the development of psychological health education in schools and the creation of better school environments.

In discussing suicide risk factors, anxiety, depression, loneliness, negative life events, despair and impulsivity are significant predictive variables for suicide (15, 16). From the perspective of the diathesis-stress models of suicide risk, adolescent suicide is not solely a response to acute stress events but is also influenced by individual qualities (17), where psychological qualities play a crucial role. Adolescence marks a period not only of rapid brain growth and development but also a sensitive phase for psychological health problems. In an era marked by the intertwining trends of “involution” and “lying flat,” adolescents face significant physical and psychological changes, academic and social pressures, family and interpersonal relationship adjustments. Once individuals encounter stressful events or setbacks, they are highly susceptible to developing stress-related emotions like pessimism, despair, which could lead to various issues such as “mental depletion,” also known as “psychological depletion” that affect psychological well-being. Previous studies have demonstrated close associations between psychological health problems and inadequate interpersonal communication skills, high academic pressure, poor sleep quality, risky behaviors, non-suicidal self-injury, and low sense of life meaning (18–22). These factors directly or indirectly impact an individual’s suicidal ideation. Despite the implementation of numerous policies aimed at improving mental health in China, the overall psychological health level among Chinese adolescents remains concerning, with even a trend of decline and “juvenilization” (9, 23, 24). This trend may be one of the reasons contributing to the rising suicide rates among Chinese adolescents. Therefore, due to the preventability and early intervention potential of suicide, adolescent psychological health problems demand more timely attention. Failure to address these issues promptly could result in tragedies akin to ‘the old burying the young’ occurring more frequently.

Existing research primarily focuses on a variable-centered perspective, using assessment scores to gage levels of mental health, assuming homogeneity among samples, which is not always the case. Not only do differences exist in the quantity between samples but also in their quality. To better understand the specificity of psychological health and its related characteristics among middle school students, this study employs Latent Profile Analysis (LPA), a person-centered method, to unveil heterogeneity among individuals. Previous studies have used this method to explore the latent categories of psychological health, labeling them as the “Health Group,” “Distress Group” and “Risk Group” based on dimensional scores (25). However, the studies discussed freshmen at the university level, with minimal research focused on middle school students, necessitating further clarification. Both junior and senior high school students are in the critical adolescent period of maximum physical and mental changes; however, they differ in psychological maturity, developmental patterns, and academic pressures (20). Hence, it is necessary to separately examine the psychological health status of these two groups to gain a more accurate and comprehensive understanding of developmental trends across the middle school stage.

Building on this foundation, our study primarily aims to explore the following key issues. Firstly, we seek to explore the latent categories of psychological health among students at different educational stages, aiming to reveal both commonalities and differences. Secondly, we aim to examine the influence of demographic variables on latent categories of psychological health, investigating individual factors affecting psychological health. Lastly, we aim to investigate the relationship between psychological health and suicidal ideation, uncovering the extent to which psychological health affects its emergence. This study supplements research on the psychological health of middle school students from a person-centered perspective, aiming to provide targeted and tailored psychological counseling based on the needs and issues of different groups, thereby enhancing mental health levels and reducing suicidal risk.

## 2 Methods

### 2.1 Participants

All students from grades 7 to 12, encompassing both junior high school (grades 7–9) and senior high school (grades 10–12), at a high school in Yunnan Province, China, were included in this study, totaling 1,992 students. According to the formula by a previous study *N* = (Z^2 *P*(1-*P*))/*E*^2 (26), where *N* represents the required sample size, *Z* corresponds to the value 1.96 for a 95% confidence interval (CI), *E* is the margin of error (0.03), and P is the probability value of 0.189 (based on a meta-analysis indicating an overall detection rate of 18.9% for psychological health among Chinese students) (14), the sample size required was calculated as 655. The sample size in this study meets the requirements. Our study obtained verbal consent from the participants and their parents. At the same time, our study was approved by the Ethics Committee of our host institutions (Number: YNNUPSY2022001) and the work was complied with the Declaration of Helsinki.

### 2.2 Research design

The study conducted a cross-sectional assessment of the psychological health status, suicidal ideation, and related factors among middle school students (both junior and senior high school students) using a self-report method. Data from 1992 middle school students were collected through an offline survey. Latent profile analysis initially identified psychological health subtypes among students at different educational stages. Subsequently, regression analysis was employed to examine the predictive factors, followed by an investigation into the impact of different psychological health groups on suicidal ideation. These methodologies enabled the analysis of the heterogeneity and the degree of influence on the psychological health of middle school students.

### 2.3 Procedure for data collection

The survey administrators of this study are master’s degree students majoring in psychology. They underwent standardized training conducted by teachers from the Mental Health Education Center at Yunnan Normal University before the formal testing phase. Using a standardized questionnaire at the class level, collective assessments were conducted with on-site retrieval. Each class was assigned 1–2 administrators who read the instructions to clarify the purpose of the test, the answering method, and the voluntary principle. Participants were instructed to independently complete the survey based on their individual circumstances within approximately 20 min. Data collection took place on November 29, 2022.

The exclusion criteria of questionnaire data were (1) individuals diagnosed with mental disorders; (2) incomplete questionnaire submissions; (3) patterned responses (e.g., consistently selecting “Not at all” for every item in the SCL-90) and (4) a concealment dimension score exceeds 4.

After screening and excluding invalid data, 1,944 valid questionnaires were collected, resulting in an effective rate of 97.59%. Among the participants, there were 1,079 junior high school students (Mage = 13.28, SD = 0.94, range of 11 to 17 years) and 865 senior high school students (Mage = 16.23, SD = 0.94, range of 14 to 19 years). The distribution of subjects is presented in Table 1.

**Table 1:** Distribution of subjects.

Variable	Variable Characteristics	Grade	
		Junior high school *N* (%)	Senior high school *N* (%)
Gender	Male	583 (54.03)	392 (45.32)
	Female	496 (45.97)	473 (54.68)
Place of origin	City	942 (87.30)	733 (84.74)
	Countryside	137 (12.70)	132 (15.26)
Only-children	No	256 (23.73)	283 (32.72)
	Yes	823 (76.27)	582 (67.28)
Subjective family atmosphere	Good	700 (64.87)	521 (60.23)
	Medium	332 (30.77)	305 (35.26)
	Poor	47 (4.36)	39 (4.51)

### 2.4 Measures

#### 2.4.1 Psychological state evaluation

The Symptom Checklist-90 (SCL-90) was developed by the American psychologist L.R. Derogatis in 1975 and translated and revised into Chinese by Dr. Wang (27). Widely used in China, this scale is an effective tool for assessing psychological health status and exhibits good reliability and validity. The scale consists of 90 items (i.e., “Feeling critical of others” and “Temper outbursts that you could not control.”) and is divided into 10 dimensions, namely: (1) somatization; (2) obsessive-compulsive; (3) interpersonal sensitivity; (4) depression; (5) anxiety; (6) hostility; (7) phobic anxiety; (8) paranoid ideation; (9) psychoticism and (10) sleep difficulties. Each item was answered on a 5-point Likert scale, ranging from 1 (“Not at all”) to 5 (“Extremely”). If the total score exceeds 160, or if any of the standardized subscale score exceeds 2, it indicates a certain level of symptomatology or psychological health issues in that factor. In this study, the Cronbach’s alpha was 0.980.

#### 2.4.2 Self-rating idea of suicide

Developed by Xia et al. in 2002, this scale has been clinically validated and exhibits good reliability and validity in assessing the degree of suicidal ideation (28). The scale comprises 26 items, such as “I am prone to tears or thoughts of crying” and “I sometimes tell lies,” organized into four dimensions: optimism, sleep, despair, and concealment. Participants should choose “yes” or “no” as the answer. A total score exceeds12 serves as the threshold for the presence of suicidal ideation. Moreover, if the score of the concealment dimension exceeds 4, it may indicate a tendency to withhold or mask information. In this study, the Cronbach’s alpha was 0.832.

### 2.5 Statistical analysis

The data entry was performed using Epidata 3.1. Descriptive statistics, correlation analyses, and common method bias checks were conducted using SPSS 26.0. Subsequently, Mplus 8.3 software was used in this study to conduct a latent profile analysis of the psychological health of middle school students and their correlations with demographic covariates and distal outcomes.

LPA was carried out using Mplus 8.3 to determine latent categories of psychological health among middle school students and model fit indices. Model fit indices included log-likelihood (LL), information evaluation criteria such as Akaike Information Criteria (AIC), Bayesian Information Criteria (BIC), sample size-adjusted BIC (aBIC), Entropy, Bootstrap Likelihood Ratio Test (BLRT), and Lo-Mendel-Rubin adjusted likelihood ratio test (LMR) (29). For AIC, BIC, and aBIC, smaller values indicate better model fit. Entropy evaluates the precision of classification, with values closer to 1 indicating more accurate classification. Significant results in LMR and BLRT suggest that k categories are superior to k-1 categories. Subsequently, the robust three-step method was used for regression model building, with latent categories as the dependent variable. This analysis aimed to explore the impact of demographic variables on the latent psychological health profiles using multinomial logistic regression models. Lastly, the DU3STEP method was employed to examine the predictive role of latent profiles on suicidal ideation.

## 3 Results

### 3.1 Test for common method deviation

Because the research data were collected in a single self-report form, common method bias may have existed (30). Thus, we employed the Harman’s single-factor test to examine the potential presence of common method bias. Exploratory factor analysis revealed a total of 19 factors with eigenvalues greater than one. The largest factor accounted for 33.29% of the variance, indicating that there wasn’t a significant issue with common method bias in this study.

### 3.2 Latent profile analysis with indicators of psychological health

To explore the latent patterns of psychological health among different stages of adolescence, latent profile models were established based on the mean values of the 10 factors of the SCL-90 for junior and senior high school students, serving as manifest indicators. Initially, a model that included one category was expanded to incorporate 2 to 5 classes, and the fit indices for various simulated models were computed. The model fit indices are presented in Table 2.

**Table 2:** Fitting index of latent profile analysis of psychological health among middle school students.

Number of Profiles	Log (L)	AIC	BIC	aBIC	Entropy	LMR (p)	BLRT (p)	Proportions min (%)
Junior high school category (*n* = 1,079)								
1-Class	−11438.45	22916.91	23016.58	22953.06				
2-Class	−7816.66	15695.31	15849.81	15751.35	0.98	0.00	0.00	0.18
3-Class	**−6332.54**	**12749.07**	**12958.39**	**12824.99**	**0.96**	**0.03**	**0.00**	**0.11**
4-Class	−5707.41	11520.81	11784.95	11616.62	0.96	0.31	0.00	0.03
5-Class	−5365.99	10859.97	11178.94	10975.66	0.93	0.14	0.00	0.03
Senior high school category (*n* = 865)								
1-Class	−8800.15	17640.29	17735.55	17672.03				
2-Class	−6247.41	12556.82	12704.47	12606.02	0.96	0.00	0.00	0.26
3-Class	−5321.55	10727.09	10927.13	10793.75	0.94	0.01	0.00	0.13
4-Class	**−4865.21**	**9836.41**	**10088.84**	**9920.52**	**0.93**	**0.01**	**0.00**	**0.06**
5-Class	−4653.13	9434.27	9739.08	9535.83	0.90	0.00	0.00	0.05

Bold indicates the best-fit model and fitting indicators. AIC, Akaike information criteria; BIC, Bayesian information criteria; aBIC, adjusted Bayesian information criteria; LMR, Lo–Mendell–Rubin likelihood ratio Test; BLRT, bootstrap likelihood ratio test.

**Figure 1:**
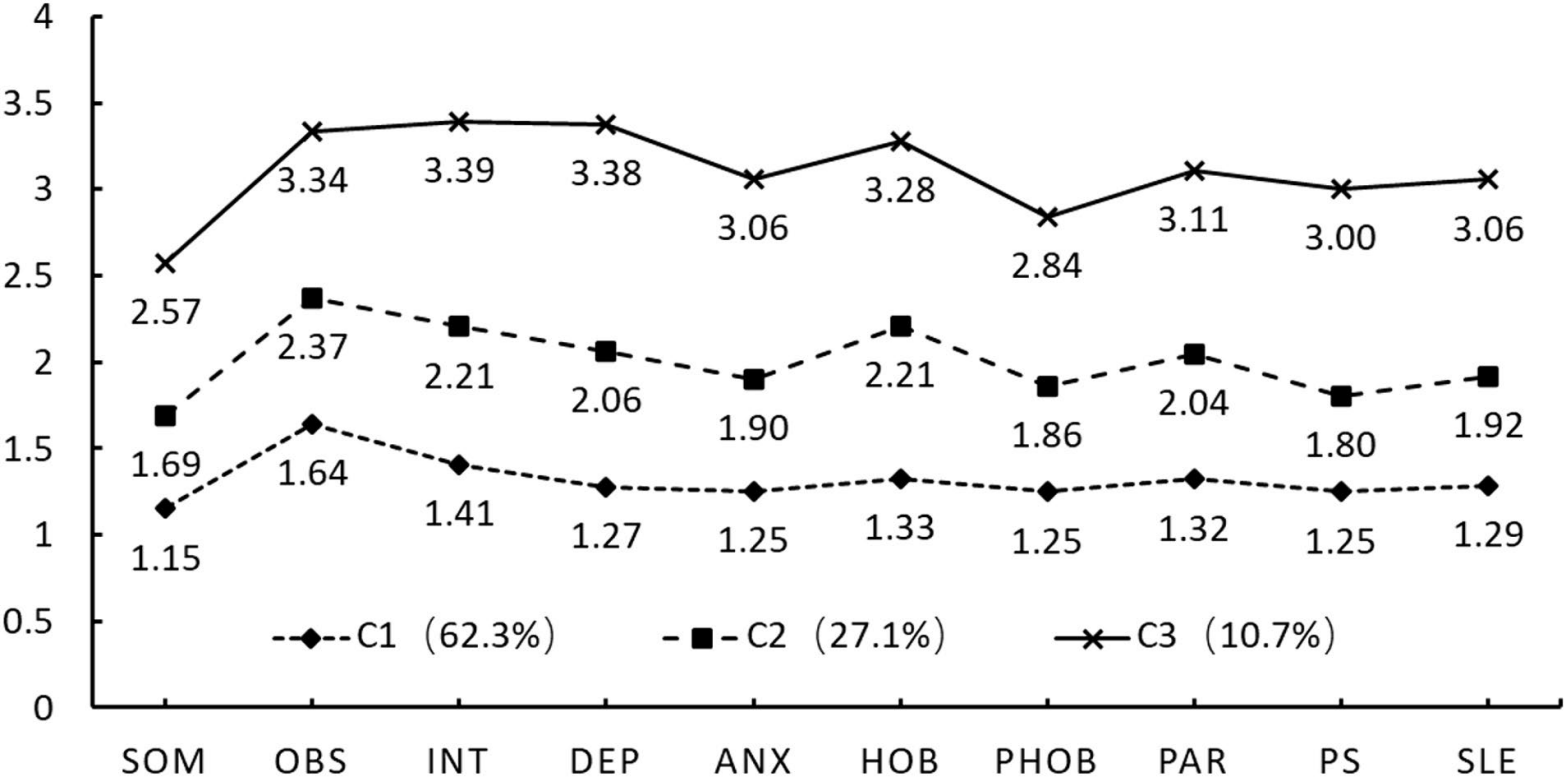
Conditional means of psychological health of junior high school student. Class1 = Psychological Health type; Class2 = Low-risk type; Class3 = High-risk type. SOM, Somatization; OBS, Obsessive-compulsive; INT, Interpersonal sensitivity; DEP, Depression; ANX, Anxiety; HOS, Hostility; PHOB, Phobic anxiety; PAR, Paranoid ideation; PSY, Psychoticism; SLE, Sleep difficulties.

The results revealed that among the junior high school group, the fit indices AIC, BIC, and aBIC decreased as the number of categories increased, with the rate of reduction slowing after reaching the category 3. The Entropy value was 0.96, indicating a classification accuracy exceeding 90%, and both LMR and BLRT demonstrated significant *p*-values. However, in category 4, LMR was not significant, and the proportion of individuals in one category was less than 5%, suggesting inadequate typicality and classification reliability (31). Furthermore, the probability of the 3-profile solution model being assigned to their respective categories exceeded 97%. In summary, the 3-profile model has determined as the optimal model for junior high school students, with mean conditions shown in Figure 1. As depicted in Figure 1, there are no intersections between the three subtypes of psychological health dimensions among junior high school students showed no intersections between dimensions, and different subtypes exhibited relatively consistent trends. The mean values of psychological health dimensions in C1 ranged from 1.15 to 1.64, indicating higher psychological health levels compared to other categories and named Psychological Health type, accounting for 62.3% of the sample. C2 had mean values in psychological health dimensions ranging from 1.69 to 2.37, with an overall mean of 2, termed as Low-risk type, comprising 292 individuals, accounting for 27.1%. C3 had mean values in the psychological health dimensions ranging from 2.57 to 3.39, with an overall mean of 3.1, labeled as High-risk type, comprising 115 individuals, accounting for 10.7%.

**Figure 2:**
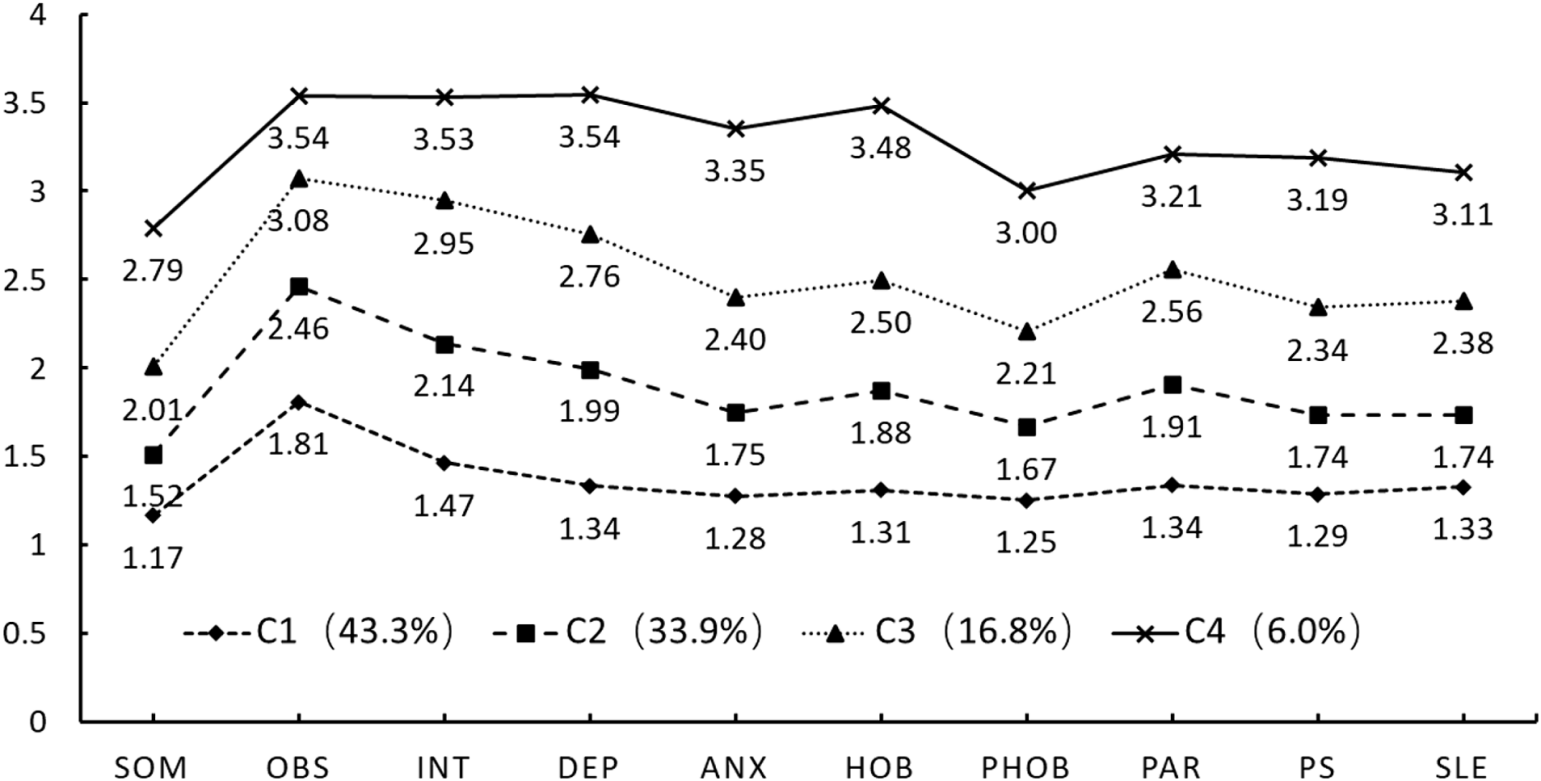
Conditional means of psychological health of senior high school student. Class1 = Psychological Health type; Class2 = Low-risk type; Class3 = Medium-risk type; Class4 = High-risk type. SOM, Somatization; OBS, Obsessive-compulsive; INT, Interpersonal sensitivity; DEP, Depression; ANX, Anxiety; HOS, Hostility; PHOB, Phobic anxiety; PAR, Paranoid ideation; PSY, Psychoticism; SLE, Sleep difficulties.

In the senior high school group, the rate of decrease in the model fitting index slows significantly when reaching category 4. Simultaneously, the Entropy was 0.93, and the LMR and BLRT were statistically significant, with probabilities of classification to respective categories exceeding 95%. Therefore, the 4- profile model was chosen as the optimal model for senior high school students. As shown in Figure 2, C1, C2, C3, and C4 exhibited varying levels across dimensions, similar to the junior high school group but with an additional category. Hence, the categories C1 (43.3%), C2 (33.9%), and C4 (6.0%) were named Psychological Health type, Low-risk type, and High-risk type, respectively. Given that the mean score of C3 (16.8%) falls between C2 and C4, it is labeled as Medium-risk type.

### 3.3 Analysis of the characteristics of each latent profile model of psychological health

The R3STEP command using the robust three-step method was employed to conduct multinomial logistic regression analysis, with LAP classification results as the outcome variable and gender, place of origin, only child status, subjective family atmosphere and age as independent variables. The relationship between demographic variables and different latent psychological health categories was determined based on Odds Ratios (OR) coefficients and their significance. Two regression models were established for junior high school and senior high school students, with the Psychologically Healthy type serving as the reference group for each model. The results are presented in Table 3.

**Table 3:** Multinomial logistic regression of latent classes of psychological health among different school segments.

Junior High School (*n* = 1,079)		Low-risk type (C2)						High-risk type (C3)		
		*B*	SE	OR				*B*	SE	OR
Gender	Female	0.53	0.15**	1.70				0.72	0.22**	2.06
	Male			1.00						1.00
Place of origin	City	−0.20	0.41	0.82				−0.36	0.52	0.70
	Countryside			1.00						1.00
Only-children	No	0.27	0.18	1.31				0.26	0.27	1.29
	Yes			1.00						
Subjective family atmosphere	Poor	1.01	0.16***	2.73				1.66	0.24***	5.24
	Medium	2.00	0.47***	7.37				3.49	0.47***	32.74
	Good			1.00						1.00
Age		−0.04	0.08	0.97				−0.01	0.11	1.00
Senior High School (*n* = 865)		Low-risk Type (C2)			Medium-risk Type (C3)			High-risk Type (C4)		
		B	SE	OR	B	SE	OR	B	SE	OR
Gender	Female	1.64	0.41***	5.17	0.27	0.18	1.31	0.70	0.22**	2.01
	Male			1.00			1.00			1.00
Place of origin	City	0.39	0.43	1.48	0.08	0.22	1.08	0.32	0.28	1.38
	Countryside			1.00			1.00			1.00
Only-children	No	0.28	0.36	1.33	0.21	0.19	1.24	−0.22	0.23	0.80
	Yes			1.00			1.00			1.00
Subjective family atmosphere	Poor	1.35	0.34***	3.87	0.86	0.19***	2.35	1.71	0.23***	5.52
	Medium	2.36	0.72**	10.57	1.69	0.56**	5.44	2.60	0.56***	13.44
	Good			1.00			1.00			1.00
Age		−0.36	0.20	0.70	−0.10	0.09	0.90	−0.07	0.12	0.93

**p < 0.01. ***p < 0.001. B, logit estimation; SE, standard errors; OR, Odds Ratio.

Gender and subjective family atmosphere significantly predicted the latent psychological health categories in both the junior high school and senior high school groups. However, other demographic factors showed no significant effects. Specifically, concerning gender, compared to males, females were more likely to be categorized into the Low-risk type and High-risk type in both junior high school and senior high school groups. In terms of the subjective family atmosphere, students reporting medium or poor subjective family atmospheres were more likely to experience psychological health issues compared to those with a good family atmosphere.

### 3.4 Comparison of suicidal ideation levels among different psychological health subtypes

The DU3STEP command using the robust three-step method was employed to investigate the relationship between different psychological health subtypes and suicidal ideation, with LAP analysis results as the independent variable and suicidal ideation level as the dependent variable.

As shown in Table 4. The data revealed significant overall differences in suicidal ideation scores among different latent psychological health categories for both junior high school and senior high school students (*χ*^2^ = 1178.71, 1174.85, *p* < 0.001), with significant differences observed between each pair of categories. Comparative analysis showed that among junior high school students, those classified as High-risk type (C3) had the highest suicidal ideation scores, followed by the Low-risk type (C2), while the Psychological Health type (C1) exhibited the lowest suicidal ideation scores. For senior high school students, the suicidal ideation scores ranked as High-risk type (C4) > Medium-risk type (C3) > Low-risk type (C2) > Psychological Health type (C1), with statistically significant differences between all groups (*p*<0.001).

**Table 4:** Test for differences in suicidal ideation in potential categories of psychological health categories among middle school students.

Suicidal Ideation	Type	M ± SE	Type comparison	*χ* ^2^
Junior high school group (*n* = 1,079)	1 Psychological Health	2.70 ± 0.11	Overall test	1178.71***
	2 Low-risk	8.36 ± 0.24	1 vs 2	440.64***
	3 High-risk	14.24 ± 0.38	1 vs 3	864.56***
			2 vs 3	170.95***
Senior high school group (*n* = 865)	1 Psychological Health	2.99 ± 0.13	Overall test	1174.85***
			1 vs 2	225.48***
	2 Low-risk	6.98 ± 0.22	1 vs 3	538.64***
			1 vs 4	641.21***
	3 Medium-risk	11.14 ± 0.31	2 vs 3	115.75***
			2 vs 4	248.95***
	4 High-risk	14.86 ± 0.45	3 vs 4	45.49***

***p < 0.001. M, mean; SE, standard errors.

## 4 Discussion

### 4.1 Analysis of latent psychological health categories and heterogeneity

Our study identified through LPA analysis heterogeneity in the psychological health status of junior high school and senior high school students. The psychological health of junior high school students was categorized into three types: Psychological Health type (62.3%), Low-risk type (27.1%) and High-risk type (10.7%). For senior high school students, psychological health was classified into four types: Psychologically Healthy type (43.3%), Low-risk type (33.9%), Medium-risk type (16.8%) and High-risk type (6.0%). Overall, the distribution of these types resembles a pyramid shape, with the psychological health type comprising the largest proportion, indicating that most students had good psychological health. However, the classification results for junior high school students are similar to those of Dong et al. (25), while the psychological health status of senior high school students is more diverse. The scores of the “High-risk type” among senior high school students are higher than those of the same category in the junior high school group, and the appearance of the “Medium-risk type” category is notable. Despite both being adolescents, junior high school and senior high school students exhibit significant differences in many aspects (32). It is noteworthy that Glenn et al.’s review also found that the global suicide rate among older adolescents (15–19 years old) is higher than that of younger adolescents (10–14 years old). Furthermore, the presence of the “Medium-risk type” among senior high school students may be attributed to several factors. Firstly, junior high school students exhibit lower psychological maturity, rapid but unbalanced psychological development, and are prone to polarized emotions, which may lead to rapid changes in psychological issues. Secondly, senior high school students are in a contradictory period of self-identity and role confusion, where various complex psychological problems may emerge during the process of self-development (33). Thirdly, as the grade level increases, students face increasingly diverse and complex situations, and their ability to cope with stressful events also shows certain differentiation. Thus, the psychological health status of senior high school students tends to be more stable compared to junior high school students. In the end, our study results validate the findings of meta-analyses, indicating that psychological health problems among middle school students worsen with increasing grades (14, 34). Therefore, it is essential and meaningful for schools to develop distinct mental health curricula for junior and senior high school students, aiding both groups in their transitions to the next level (adult or college).

Post-hoc comparisons revealed consistent trends in the shape of the various dimensions within each subtype across different academic stages, with significant differences observed between subtypes. In the junior high school group, the scores for the psychological health dimensions of the High-risk type were higher than the Low-risk type, while the Low-risk type showed significantly higher scores than the psychological health type; a similar trend was evident in the senior high school group. These findings supported the conclusion that inherent heterogeneity exists in the psychological health of middle school students. These findings could provide valuable reference information for developing targeted intervention measures and enhancing psychological health literacy for students in different academic stages.

### 4.2 Impact of demographic variables on latent psychological health categories

Our study further discovered significant differences in gender and subjective family atmosphere among different academic stages psychological health subtypes. Specifically, the risk of psychological health issues was found to be higher among female students compared to males, which is consistent with previous research findings (35, 36). However, there remains no consensus in the academic community regarding gender differences in the psychological health of middle school students. A meta-analysis found that the suicide risk among adolescent males is higher than that among females (34). The significant difference observed in our study compared to the aforementioned findings can largely be attributed to cultural differences. For example, in some Western countries, individuals have the right to use firearms (whether legal or illegal), and issues such as alcohol abuse and drug use are more prevalent compared to China. Additionally, in Yu et al.’s meta-analysis (37), no significant gender differences were found in the internalizing and externalizing problems of psychological health during the junior and senior high school stages. The reasons for the discrepancies found in this study compared to the above results may include the following points. Physiologically, adolescent girls face greater pressure and confusion arising from physiological developments compared to boys (38). Additionally, the increased levels of estrogen and progesterone in females can contribute to heightened negative emotions (39). Regarding personality traits, girls’ susceptibility makes them more sensitive and delicate (40), intensifying emotional responses and making them more prone to prolonged emotional fluctuations when facing adverse events. Within the social and cultural context, females encounter a higher number of stressors such as gender-biased family traditions, workplace gender discrimination, and cultural constraints on behavior, impacting their psychological health to a greater extent (41, 42). This underscores the importance of families, schools and society paying particular attention to the psychological health status of female students and offering timely guidance to prevent further deterioration of their condition.

Family serves as a cornerstone of adolescent physical and mental development, representing a primary environment and a major source of stress for adolescents (43). According to the interpersonal theory of suicide, a poor family atmosphere increases the risk of deterioration in the mental health status of middle school students, a finding consistent with previous research (44, 45). The family is an important microsystem in adolescent development, indirectly influencing their psychological well-being (46), as parental negative emotions can influence their children (45). Adolescents experiencing a negative subjective family atmosphere tend to have strained parent–child relationships, lower levels of intimacy, reduced life satisfaction, decreased quality of interpersonal relationships and perceive decreased social support (44, 47–49), leading to increased susceptibility to psychological health issues. Prior studies have affirmed that stable, healthy, and affectionate family systems positively influence children’s growth (49, 50). Consequently, educators should pay attention to students’ family situations, intervene appropriately, and help parents effectively enhance their parenting skills, contributing collaboratively to students’ healthy development. Furthermore, the research findings underscore the importance for parents to create a warm and affectionate family atmosphere, actively engage in family education, and pay attention to their children’s physical and mental well-being.

### 4.3 The relationship between psychological health latent categories and suicidal ideation

The detection rate of suicidal ideation among junior high school students was 14.27%, higher than the detection rate of 11% among junior high school students in China from 2010 to 2020, as reported in a meta-analysis (using the same measurement tool) (32). For high school students, the detection rate of suicidal ideation was 16.65%, which is consistent with previous studies (51, 52). The suicidal ideation levels of High-risk type among junior and senior high school students were significantly higher than other category groups, aligning with conclusions drawn from a variable-centered perspective. Past research has consistently shown that individuals with lower levels of psychological well-being often experience anxiety, depression, sleep problems (37), decreased subjective well-being (53), and increased antisocial behavior (54), all of which pose significant challenges and risks to their academic performance, daily lives, and overall health, sometimes leading to suicidal behavior (55). Our study confirm previous research indicating that individuals at risk of poor psychological health are more likely to develop suicidal thoughts, leading to increased suicidal ideation (7, 56). Furthermore, compared to previous research, the detection rate of suicidal ideation among junior and senior high school students in China is relatively high compared to adolescent populations in Asia, Europe, and other regions, but lower than in Africa (57, 58). These findings underscore the persistent nature of adolescent mental health issues, emphasizing the importance of addressing these concerns despite the return to normalcy in activities and mobility following the pandemic.

As suicidal ideation stands as one of the most potent predictors of future suicidal behavior (54), it underscores the imperative to steadfastly implement the “Comprehensive Enhancement and Improvement Plan for Mental Health Work of Students in the New Era (2023) - 2025,” firmly placing mental health work in a more prominent position. It emphasizes bolstering adolescent protection, focusing on mental health education, and tailoring preventive measures according to various psychological health conditions to avoid tragic occurrences while better nurturing the younger generation for the responsibilities of national rejuvenation. Firstly, for students in the High-risk type, targeted suicide prevention measures involving evidence-based small-group psychological counseling, supplemented with individual psychological interventions and therapy, are recommended. Teachers and mental health committee members should closely monitor the safety of such students to curb the transition from suicidal ideation to actions, averting severe adverse consequences. Secondly, for students in the Medium-risk type, who display positive indicators across the 10 dimensions of psychological health, their average suicidal ideation values are close to the critical threshold. Hence, suicide prevention measures should involve medium-sized group psychological counseling, providing emotional support and humanistic care to alleviate negative emotions and extinguish psychological crises in their budding state. Thirdly, for students in the Low-risk type, despite lower scores on suicidal ideation, factors like obsession-compulsive, interpersonal sensitivity, depression, hostility, and paranoid ideation are close to or exhibit positive trends. Therefore, suicide prevention efforts should not be taken lightly; targeted dissemination of mental health programs addressing specific issues can be helpful. Lastly, for the majority of students in the Psychologically Health type, efforts should concentrate on alleviating obsessive symptoms and managing strained interpersonal relationships. Prevention measures should primarily focus on psychological health education courses, aiming to cultivate positive psychological qualities like gratitude, psychological resilience, self-efficacy, and a sense of life’s meaning to maintain individual mental health and prevent the emergence of suicidal ideation. These interventions and recommendations above will facilitate schools to help adolescents improve their mental health and effectively prevent the emergence and decline of suicide.

## 5 Limitations and future directions

The study adopted a person-centered perspective to explore the group heterogeneity of psychological health among students in different academic stages, yet it still has certain limitations.

Firstly, the research data analysis was based on a cross-sectional approach, lacking the ability to make causal inferences. Consequently, the stability or transition patterns among different subtypes of secondary students over time remain unclear. Thus, future studies could utilize a longitudinal design to establish latent transition analysis models and causal experimental inferences to enhance the findings of this study. Secondly, the measurements relied solely on self-reporting, potentially leading to a lack of clarity regarding the psychological conditions of this group and the detection rates of suicidal ideation. Future research could incorporate various methods such as parental assessments, teacher evaluations, and interviews to gather a more comprehensive understanding. Thirdly, our study was conducted in a single city in China. To improve sample coverage and representativeness, future research could expand to include other regions. Additionally, caution should be exercised when generalizing research results due to cultural differences and other factors. Fourthly, employing social network analysis methods in future investigations could explore the relationships among symptoms of suicidal ideation within the network. Utilizing indicators such as centrality, it could identify core symptoms and their intrinsic correlations, thus providing reliable intervention targets.

## 6 Conclusion

In summary, this study contributes to our understanding of the relationship between mental health and suicidal ideation among middle school students. Our empirical findings reveal significant heterogeneity in psychological health of both junior and senior high school students. Specifically, junior high school students demonstrated three latent categories of psychological health: Psychologically Health type, Low-risk type, and High-risk type, while senior high school students showed four categories: Psychologically Health type, Low-risk type, Medium-risk type, and High-risk type. Gender and subjective family atmosphere significantly influence the latent classification of psychological health among secondary school students. Female students exhibit poorer psychological health compared to males, while students with lower evaluations of family atmosphere tend to have lower levels of psychological well-being. There are significant differences in suicidal ideation scores among students in different academic stages based on their latent psychological health categories. The High-risk type exhibits the highest levels of suicidal ideation, followed by the Medium-risk type, while the Low-risk type and Psychologically Health type demonstrate comparatively lower levels of suicidal ideation.

Previous studies have tended to combine junior and senior high school students as adolescents and discuss them together, but in reality, there are significant differences in the developmental level and mental health status of these two groups, necessitating separate discussions and targeted interventions and responses. Therefore, based on the findings of this study and practical experience, we recommend that schools adopt targeted mental health education for middle school students of different age groups, and implement individualized interventions for individuals with different mental health conditions, in order to improve the effectiveness of mental health education and create a better school environment.

## Data availability statement

The raw data supporting the conclusions of this article will be made available by the authors, without undue reservation.

## Ethics statement

The studies involving humans were approved by the Ethics Committee of our host institutions (Number: YNNUPSY2022001). The studies were conducted in accordance with the local legislation and institutional requirements. Written informed consent to participate in this study was not required from the participants/the participants’ legal guardians/next of kin in accordance with the national legislation and the institutional requirements. Verbal informed consent and verbal informed assent to participate were provided by the participants legal guardian/next of kin and the participants respectively.

## Author contributions

MW: Conceptualization, Data curation, Formal analysis, Investigation, Validation, Visualization, Writing – original draft, Writing – review & editing. JL: Funding acquisition, Project administration, Resources, Supervision, Writing – review & editing.
